# The impact of COVID-19 on walking practices in Korea: Policy implications for Urban health and physical activity resilience

**DOI:** 10.1371/journal.pone.0338875

**Published:** 2025-12-19

**Authors:** Jeongsun Park, Changwoo Shon

**Affiliations:** 1 Graduate School, Inje University, Busan, Republic of Korea,; 2 Division of Health Administration, College of Software and Digital Healthcare Convergence, Yonsei University, Wonju, Republic of Korea; University of Chicago Division of the Biological Sciences, UNITED STATES OF AMERICA

## Abstract

**Background:**

This study adopts a socio-ecological model to evaluate the individual- and community-level factors influencing walking practices among urban adults before (2018−2019) and after (2020−2021) the COVID-19 pandemic. Busan, South Korea, characterized by dense urban environments and structured health promotion systems, offers a relevant context to examine pandemic-related changes in physical activity behaviors.

**Methods:**

The 2018–2021 Korea Community Health Survey was used, targeting adults aged 19 and older living in Busan Metropolitan City. Multilevel logistic regression models were constructed using data from 58,028 individuals (Level 1) and 16 administrative districts (Level 2). The binary dependent variable was walking. The independent variables included individual-level and community-level factors. Model fit was evaluated using the intraclass correlation coefficient, likelihood ratio, and −2 log likelihood.

**Results:**

Individual-level variables such as sex, age, household income, subjective health status, and depression significantly correlated with walking before and after the COVID-19 pandemic. Females who perceived their health as poor or experienced depression were less likely to walk. However, the associations between age, household income, and walking practices were inconsistent. Before the pandemic, individuals who reported high trust in their neighbors were more likely to engage in walking practices. After the pandemic, those classified as obese were less likely to engage in walking practice, while individuals who engaged in alcohol consumption showed higher odds of walking practice. Moreover, participants who perceived access to public transportation as good had increased odds of walking. At the community level, pedestrian paths and social network difficulty were negatively associated with walking practice. In contrast, the availability of public sports facilities was positively associated with walking.

**Conclusion:**

The COVID-19 pandemic significantly affected urban adults’ walking habits. Factors such as pedestrian paths, sports facilities, and reduced social relationship challenges helped maintain walking practices. These results highlight the need for multilevel interventions that target environmental, social, and individual determinants to promote and sustain walking during and after public health emergencies. In Korea and other high-density Asian cities, these findings provide policy-relevant evidence to guide walkability-oriented urban planning and health promotion strategies that strengthen physical activity resilience in future public health crises.

## Introduction

Walking is a universally accessible physical activity that can be practiced regardless of sex, age, ethnicity, socioeconomic status, or baseline health condition, making it one of the most inclusive forms of movement for urban populations [1,2]. Regular walking helps prevent and manage chronic diseases such as cardiovascular disease, diabetes, obesity, and certain cancers [3,4], improves musculoskeletal health by maintaining bone density, joint function, and muscular strength [5,6], and protects mental health by reducing stress, depression, and anxiety while enhancing emotional well-being [7,8]. Walking in green urban spaces and natural environments further strengthens psychological benefits [9,10] and fosters social interaction and neighborhood connectedness through encounters in public spaces [11]. As a low-carbon mode of transport, walking also contributes to urban sustainability by reducing car dependence, congestion, and air pollution [12], helps maintain functional independence and prevent falls among older adults [13], and may lower transport and healthcare expenditures at the population level [14,15].

Despite these well-established benefits, walking practice has declined among Korean adults, especially in the context of the COVID-19 pandemic [16,17]. In South Korea, the proportion of adults meeting the national walking recommendation decreased from 40.4% in 2019 to 37.4% in 2020 [18], and the World Health Organization reported that more than 80% of adolescents and 27% of adults worldwide failed to achieve recommended physical activity levels, a gap that widened under pandemic-related restrictions [19]. Non-pharmaceutical interventions such as social distancing, stay-at-home orders, remote working, and restrictions on public space access have substantially reduced everyday opportunities for outdoor physical activity [20], while contributing to increased obesity and mental health problems linked to physical inactivity [21]. In Korea, these trends prompted the Health Plan 2030 to designate adult walking practice as a key national health indicator and to strengthen monitoring of walking behavior [17]. At the global level, recent evidence from WHO and OECD shows that physical inactivity has remained high in the post-pandemic period. A pooled analysis of 507 population-based surveys from 197 countries estimated that about one third (31.3%) of adults worldwide did not meet WHO recommendations for physical activity in 2022, and that the global prevalence of insufficient activity has increased since 2010 [22]. In Europe, a joint WHO/OECD report indicates that more than one in three adults are insufficiently active and highlights walking and other forms of active transport as cost-effective policy levers for COVID-19 recovery [2]. These global trends underscore the importance of understanding how walking practice changed during and after the COVID-19 pandemic in specific national contexts such as Korea. In national statistics, this indicator is reported as the “walking practice rate,” based on the CHS definition of meeting the recommended threshold of ≥30 minutes of walking per day on ≥5 days per week. Throughout this paper, we therefore use the term *walking practice* to denote this CHS-based indicator, which is conceptually equivalent to walking behavior in the international literature.

The socio-ecological model (SEM) provides a useful framework for interpreting these changes by emphasizing that health behaviors result from the dynamic interactions among intrapersonal, interpersonal, organizational, community, and policy-level factors [23]. Applying SEM to walking, prior studies in Korea and other countries have shown that individual characteristics such as age, sex, educational attainment, household income, and health behaviors (e.g., smoking, alcohol use) are consistently associated with walking practice [24–26]. Interpersonal conditions, including marital status and cohabitation, as well as organizational and community features—such as neighborhood safety, social trust, and perceived access to public transportation—also shape walking behavior by influencing perceived support and barriers in daily life [27–29].

At the environmental level, a growing body of research has highlighted how urban design, land-use mix, pedestrian infrastructure, and availability of recreational facilities are linked to physical activity and walking [1,27,30]. Korean studies drawing on community surveys and geographic information systems (GIS) have reported that neighborhood parks, sidewalks and trails, and community sports facilities are positively associated with walking among adults and older adults [29,31,32]. During the COVID-19 pandemic, international evidence further indicated that changes in walking were closely tied to local built environments and restrictions: outdoor recreational walking increased in some cities, whereas utilitarian walking and overall step counts declined, particularly in disadvantaged areas [33–36]. However, relatively few studies have simultaneously examined individual- and community-level determinants of walking before and after the COVID-19 pandemic using a unified analytic framework, and evidence from East Asian urban contexts remains limited.

Busan Metropolitan City offers a particularly relevant setting for investigating multilevel determinants of walking in the context of COVID-19. As South Korea’s second-largest city, Busan has implemented pedestrian-friendly and gender-sensitive urban planning policies, including the long-standing “City of Walking” initiative and the designation of multiple women-friendly districts [37,38]. Despite these efforts, recent Community Health Survey results show a downward trend in adult walking practice in Busan [39], prompting the city to adopt a “15-minute city” strategy that aims to ensure access to essential services within walking distance [40]. This policy context underscores the need for empirical evidence on how individual characteristics, perceived neighborhood conditions, and objectively measured community resources jointly influence walking behavior during and after a major public health crisis.

Building on this literature and policy context, the present study applies a socio-ecological perspective to examine changes in walking practice among urban adults in Busan before (2018–2019) and after (2020–2021) the COVID-19 pandemic. Using multilevel logistic regression, we link four consecutive waves of the Korea Community Health Survey to district-level indicators of urban parks, pedestrian paths, public and private sports facilities, and social network difficulty. This study contributes to the extant literature on walking and urban health in three main ways. First, to our knowledge, it is the first study in Korea to compare determinants of walking practice before and after the COVID-19 pandemic within a unified multilevel framework grounded in the socio-ecological model. Second, by combining individual health behaviors and subjective neighborhood perceptions with objective indicators of the built and social environment, it provides a more comprehensive assessment of community determinants than previous studies that relied mainly on self-reported environmental measures. Third, by focusing on Busan—a dense metropolis that has implemented “City of Walking” and “15-min city” initiatives—the study generates context-specific yet transferable evidence on how urban environments and social conditions can support walking and physical activity resilience during and after public health emergencies. The findings are expected to inform urban health policies and walkability-centered interventions designed to enhance physical activity resilience in future crises.

## Materials and methods

### Data source

The relationship between physical and social environmental factors influencing walking practices among adults was evaluated using individual-level data for 2018–2021, collected from the raw Community Health Survey (CHS), an officially recognized national statistic approved by Statistics Korea (Approval No. 11775). Raw data were obtained from the Korea Disease Control and Prevention Agency (KDCA) through official request with prior approval. In the CHS, a stratified cluster sampling method was applied to select approximately 900 adults aged 19 or older per public health center. Each public health center forms a field survey team in collaboration with a designated local university that collects the data. Trained interviewers visited the households by surveying one respondent per household and all members aged 19 or older. Data were collected through face-to-face interviews using Computer-Assisted Personal Interviewing technology [41].

Community-level data were sourced from (1) Busan Metropolitan City Basic Statistics (Urban Parks), (2) the Busan Galmaet-gil database and National Pedestrian Path Information Standard Dataset for pedestrian path data, (3) the Ministry of Culture, Sports and Tourism’s National Public Sports Facility Database for public sports facilities, (4) Busan Metropolitan City’s Registered/Reported Sports Facilities Database for private sports facilities, and (5) the Busan Community Welfare Survey for social relationship difficulties. Additional community-level information was obtained from the Korean Statistical Information Service and the Busan Public Data Portal (Big Data Wave). Unlike previous Korean studies on walking that relied solely on individual-level survey data, this study linked CHS microdata with multiple administrative and open-government datasets to construct district-level indicators of parks, pedestrian paths, sports facilities, and social network difficulty.

Inclusion criteria included adults aged 19 or older residing in Busan Metropolitan City. After excluding 14 cases with missing values or non-responses, the final sample comprised 58,028 individuals (2018–2019: *n* = 29,018; 2020–2021: *n* = 29,010). The data were structured across 16 administrative regions.

### Study variables

The dependent variable was walking practice, defined by the KDCA as walking for at least 10 min per session, for 30 min or more per day, on five or more days in the past week, and assessed based on the following questions: “During the past week, on how many days did you walk for at least 10 min at a time?” and “On one of these days, how long did you usually walk?” Responses were classified as “yes” or “no” for walking practice, including commuting, transportation, and exercise. In the CHS and national reports, this indicator is labelled “walking practice” and its prevalence is reported as the “walking practice rate.” Conceptually, this indicator corresponds to walking behavior that meets the national recommendation for health benefits; therefore, throughout this paper, we use the term walking practice to refer to this CHS-based walking behavior indicator. The independent variables included individual- and community-level factors influencing walking practice, selected based on the SEM of environmental influences on physical activity [24,28].

#### Individual-level factors.

The sociodemographic factors included sex, age, education level, household income, and marital status. Sex was treated as a binary variable (male versus female). Age was categorized into five groups: 19–34, 35–49, 50–64, 65–74, and ≥75, based on the respondent’s birthdate. Education level was categorized as junior high school, high school, or university. Household income was measured in response to the following question: “What was your household’s total income over the past year, including all sources, such as wages, real estate income, pensions, interest, government subsidies, and monetary support from relatives or children? Please provide the monthly average if the annual amount cannot be estimated.” The responses were collected as continuous variables in units of 10,000 KRW and converted to monthly household income. This was then categorized into income quintiles: first (lowest 20%), second (lower 40%), third (lower 60%), fourth (upper 40%), and fifth (highest 20%). Marital status was assessed by asking, “What is your current marital status (including common-law marriage)?” The response options included currently married (cohabiting), divorced, widowed, separated, and never married. For analysis, the responses were grouped into two categories: partnered and single.

Healthy behavior and health status factors included subjective health status, obesity, depression, number of chronic diseases, smoking, and alcohol consumption. Subjective health status was assessed using the question, “How would you describe your usual health condition?” Responses were categorized as “good” (very good or good) or “poor” (fair, poor, or very poor). Obesity was defined by a body mass index (BMI) of 25 kg/m2 or higher, calculated as weight (kg) divided by the square of height (m). Following the criteria established by the World Health Organization Western Pacific Regional Office and the Korean Society for the Study of Obesity, individuals with a BMI of 25 kg/m2 or higher were classified as “yes,” and those with a BMI below 25 kg/m2 were classified as “no” [42,43]. Depression was assessed using the question, “During the past year, have you felt sadness or despair that interfered with your daily life for two weeks or longer?” Responses were categorized as “yes” or “no.” Several chronic diseases were assessed using responses to the questions, “Have you ever been diagnosed with hypertension by a physician?” and “Have you ever been diagnosed with diabetes by a physician?” Based on the responses, participants were categorized into “0,” “1,” or “above 2” chronic conditions. The following question assessed smoking status: “Do you currently smoke cigarettes?” and categorized it as “yes” or “no.” Alcohol consumption was assessed using the question, “How often do you drink alcohol?” and categorized as “yes” or “no.”

Factors influencing individuals’ subjective perceptions of their communities included trust among neighbors, neighborhood safety, the natural environment, and access to public transportation [25]. Subjective perceptions of a community refer to individuals’ evaluations of their physical and social environments. In this study, the following items were used to measure this variable: (1) “People in my neighborhood trust and rely on each other,” (2) “I am satisfied with the overall safety of my neighborhood (e.g., natural disasters, traffic accidents, farming-related injuries, crime),” (3) “I am satisfied with the natural environment of my neighborhood (e.g., air quality, water quality),” and (4) “I am satisfied with the public transportation conditions in my neighborhood (e.g., buses, taxis, subways, trains).” Responses to these questions were categorized as binary variables: “good” or “poor.”

#### Community-level factors.

Community-level factors included urban parks [24], pedestrian paths [31], public sports facilities [26,29], private sports facilities [32], social network difficulty [24,26,36]. Urban parks were defined as urban park areas. Urban parks consisted of residential and neighborhood parks (historical, cultural, waterside, cemetery, sports, and urban agricultural parks). Data from the Busan Metropolitan City Basic Statistics (Parks) [44] were used.

Pedestrian paths included promenades and pedestrian-only roads. A pedestrian path was a designated area for pedestrian passage, while promenades were intended for walking for exercise or enjoyment (e.g., trails, neighborhood alleys, historical site trails, and forest paths). Data from Busan Galmaet-gil and Busan Downtown Galmaet-gil of Busan’s walking paths [38] were used. The promenade data were sourced from the National Road Tourism Information Standard Data [45], and the pedestrian-only roads data were obtained from the Urban Planning Status [46]*.*

Data on the status of village sports facilities among public sports facilities nationwide were used to assess public sports facilities, i.e., public physical activity facilities [47]. This included several simple sports facilities such as soccer, basketball, volleyball, badminton, tennis, gateball, and community gyms. Data on the status of private sports facilities, i.e., private physical activity facilities, were obtained from Busan Metropolitan City’s reported or registered sports facilities [48]. These included comprehensive sports facilities, swimming pools, gyms, golf driving ranges, physical training centers, and ice rinks.

Data regarding social networking difficulties were obtained from the Busan Community Social Security Survey [49] and included the extent of the problems experienced in social relationships, such as conflicts with relatives or neighbors, feelings of isolation or disconnection, and difficulties within affiliated groups. These difficulties were measured based on the question, “In the past year, have you experienced any of the following difficulties in social relationships?” Responses were recorded on a five-point Likert scale ranging from “Never experienced” (1 point) to “Very frequently experienced” (5 points). The responses represented continuous variables, with higher scores indicating more significant difficulties with social networks. The reference points for the community-level factors were 2019 for the period before COVID-19 and 2021 for the period after COVID-19.

The definitions, coding, and measurement levels of all variables included in the models are summarized in S1 Table.”

### Analysis

The study data were structured into two levels: 1 (individual) and 2 (community). The analysis included data from 58,028 individuals at the individual level and 16 districts at the community level. Descriptive statistics and frequency analyses were used to examine the general characteristics of the participants and measured variables. Chi-square tests were performed to assess group differences based on sociodemographic characteristics. The community-level variables were examined using descriptive statistics. Multilevel analysis was conducted to examine the association between walking practices and COVID-19.

Guided by the socio-ecological model, individuals (Level 1) were conceptualized as nested within 16 administrative districts (Level 2). To account for this hierarchical structure and the correlation of observations within districts, we used multilevel (mixed-effects) logistic regression models with a random intercept for districts and fixed effects for all individual- and community-level covariates [50]. This approach estimates the effects of Level 1 and Level 2 variables simultaneously while partitioning the variance in walking practice into within- and between-district components, rather than simply entering district characteristics into a single-level regression equation. Four nested models were fitted separately for the pre- and post-COVID-19 periods: Model 1 (null model with random intercept only), Model 2 (Model 1 plus individual-level predictors), Model 3 (Model 1 plus community-level predictors), and Model 4 (Model 1 plus both individual- and community-level predictors). Model fit and the added value of multilevel modeling over single-level logistic regression were evaluated using the intraclass correlation coefficient, likelihood-ratio tests, and −2 log likelihood statistics.

In multilevel analyses, models are typically categorized into null and explanatory models. The null model included only a random intercept and no explanatory variables, estimating the proportion of variance in the dependent variable due to between-group differences (e.g., across communities).

A four-model multilevel framework was adopted to examine the effects of individual- and community-level variables on walking behavior. Model 1 (null model) included no independent variable and served as the baseline. Model 2 incorporated individual-level predictors to assess the independent effects of these variables. Model 3 included only community-level predictors; Model 4 combined individual- and community-level variables to evaluate their joint effects on walking practices. The model fit was evaluated using the intraclass correlation coefficient (ICC), likelihood ratio (LR), and the −2 log likelihood (−2LL). In addition, the variance inflation factor for all variables ranged from 3.19 (Public sports facilities) to 1.04 (depression), which is below the commonly accepted threshold of 10, suggesting that multicollinearity was unlikely to distort the analytical results.

#### Model diagnostics and sensitivity analysis.

In addition to the main multilevel logistic regression models, we fitted a single-level logistic regression model with the same set of covariates (sex, age, education level, household income, marital status, subjective health, obesity, depression, number of chronic diseases, smoking, alcohol consumption, trust among neighbors, perceived neighborhood safety, natural environment, access to public transportation, and five continuous community-level variables urban parks, pedestrian paths, public sports facilities, private sports facilities, and social network difficulty) in order to examine model diagnostics. Using the predict command in Stata, we obtained standardized Pearson residuals, Pregibon leverage values, and influence statistics (dx2, ddeviance, and dbeta). Observations with extreme residuals or high leverage/influence were flagged as potentially influential and were excluded in a sensitivity analysis. When we re-estimated the model after excluding these 426 observations, the overall model fit improved slightly, but the magnitude and statistical significance of the estimated odds ratios changed only marginally compared with the full-sample model (for example, the odds ratio for female compared with male changed from 0.85 to 0.84, with both estimates remaining highly significant, p < .001). These findings indicate that our results are robust to the presence of influential observations.

In logistic regression, continuous covariates are assumed to have a linear relationship with the logit of the outcome. We therefore examined the linearity-in-the-logit assumption for all continuous variables (urban parks, pedestrian paths, public sports facilities, private sports facilities, and social network difficulty). Specifically, we compared a model including only linear terms for these variables with an alternative model that additionally included quadratic terms (x2) using likelihood-ratio tests. We also tested the joint significance of each quadratic term using Wald tests. As none of the quadratic terms significantly improved model fit at the 5% level, the linear specification was retained for all continuous covariates. In a sensitivity analysis, models using categorized forms of these variables (quartiles) produced odds ratios that were very similar to those of the main analysis (data not shown).

The Community Health Survey (CHS) provides household and individual sampling weights based on its stratified cluster sampling design. While these weights are available, the present study did not apply sampling weights in the regression analyses. This decision followed previous multilevel studies using CHS data that employed unweighted models to estimate associations at both the individual and community levels. Given that the study aims to identify relational effects rather than produce population estimates, unweighted multilevel logistic regression was deemed appropriate. Nevertheless, the availability of CHS sampling weights has been acknowledged for transparency.

### Ethical review and approval

This study was conducted in accordance with the Declaration of Helsinki. It used secondary, anonymized data from the Korea Community Health Survey (CHS). The raw CHS data were requested through, and approved by, the Korea Disease Control and Prevention Agency’s Community Health Survey data portal. The study was exempt from ethical review by the Institutional Review Board (IRB) of Inje University on October 24, 2024 (IRB No. INJE 2024-10-038).

## Results

### General characteristics of participants

The general characteristics of the study participants are provided separately for the before COVID-19 (2018–2019) and after COVID-19 (2020–2021) periods (Table 1).

**Table 1:** General characteristics of study participants before and after the COVID-19 pandemic.

Variable		Before COVID-19 (2018–2019)						After COVID-19 (2020–2021)					
**Male**		**Female**		**Total**		**Male**		**Female**		**Total**	
***n***	**%**	***n***	**%**	***n***	**%**	***n***	**%**	***n***	**%**	***n***	**%**
Total		12,559	100.0	16,459	100.0	29,018	100.0	12,750	100.0	16,260	100.0	29,010	100.0
Dependent variable													
Walking	Yes	6,943	55.3	8,662	52.6	15,605	53.8	6,114	48.0	6,747	41.5	12,861	44.3
practice	No	5,616	44.7	7,797	47.4	13,413	46.2	6,636	52.1	9,513	58.5	16,149	55.7
Socio-demographic factors													
Age (years)	19–34	2,368	18.9	2,683	16.3	5,051	17.4	2,376	18.6	2,745	16.9	5,121	17.7
	35–49	3,014	24.0	3,766	22.9	6,780	23.4	3,065	24.0	3,530	21.7	6,595	22.7
	50–64	3,609	28.7	5,074	30.8	8,683	29.9	3,639	28.5	4,953	30.5	8,592	29.6
	65–74	2,196	17.5	2,856	17.4	5,052	17.4	2,238	17.6	2,990	18.4	5,228	18.0
	≥ 75	1,372	10.9	2,080	12.6	3,452	11.9	1,432	11.2	2,042	12.6	3,474	12.0
Education level	Junior high school	2,755	22.0	5,885	35.8	8,640	29.8	2,533	19.9	5,356	33.0	7,889	27.2
	High school	4,051	32.3	4,519	27.5	8,570	29.5	4,056	31.8	4,478	27.6	8,534	29.4
	University	5,748	45.8	6,049	36.8	11,797	40.7	6,154	48.3	6,420	39.5	12,574	43.4
Household income	Q1	2,059	18.0	3,522	23.3	5,581	21.0	2,341	21.6	3,761	26.8	6,102	24.5
	Q2	2,314	20.2	3,195	21.1	5,509	20.7	2,161	20.0	2,876	20.5	5,037	20.2
	Q3	2,577	22.5	3,020	19.9	5,597	21.0	1,952	18.0	2,250	16.0	4,202	16.9
	Q4	2,644	23.1	3,211	21.2	5,855	22.0	2,592	24.0	3,084	21.9	5,676	22.8
	Q5	1,858	16.2	2,202	14.5	4,060	15.3	1,775	16.4	2,090	14.9	3,865	15.5
Marital status	Partnered	8,611	68.6	9,719	59.1	18,330	63.2	8,088	63.5	9,062	55.7	17,150	59.1
	Single	3,942	31.4	6,734	40.9	10,676	36.8	4,660	36.6	7,195	44.3	11,855	40.9
Healthy behavior and health status factors													
Subjective health status	Good	4,768	38.0	5,105	31.0	9,873	34.0	6,193	48.6	6,366	39.2	12,559	43.3
	Poor	7,790	62.0	11,354	69.0	19,144	66.0	6,557	51.4	9,893	60.9	16,450	56.7
Obesity	Yes	4,362	35.3	3,847	24.1	8,209	29.0	4,662	36.6	3,702	22.9	8,364	28.9
	No	8,008	64.7	12,116	75.9	20,124	71.0	8,073	63.4	12,486	77.1	20,559	71.1
Depression	Yes	647	5.2	1,434	8.7	2,081	7.2	668	5.2	1,412	8.7	2,080	7.2
	No	11,911	94.9	15,024	91.3	26,935	92.8	12,078	94.8	14,847	91.3	26,925	92.8
Number of chronic diseases	0	8,617	68.6	11,781	71.6	20,398	70.3	8,828	69.2	11,548	71.0	20,376	70.3
	1	3,044	24.2	3,659	22.2	6,703	23.1	3,021	23.7	3,724	22.9	6,745	23.3
	Above 2	897	7.1	1,016	6.2	1,913	6.6	901	7.1	985	6.1	1,886	6.5
Smoking	Yes	4,369	34.8	524	3.2	4,893	16.9	4,160	32.6	469	2.9	4,629	16.0
	No	8,190	65.2	15,935	96.8	24,125	83.1	8,588	67.4	15,791	97.1	24,379	84.0
Alcohol consumption	Yes	8,698	69.3	6,784	41.2	15,482	53.4	7,857	61.6	5,605	34.5	13,462	46.4
	No	3,861	30.7	9,674	58.8	13,535	46.7	4,893	38.4	10,655	65.5	15,548	53.6
Subjective perception of the community factors													
Trust among neighbors	Good	3,711	61.9	5,342	66.0	9053	64.2	3,907	62.4	5,085	64.4	8,992	63.5
	Poor	2,282	38.1	2,758	34.1	5040	35.8	2,350	37.6	2,817	35.7	5,167	36.5
Perceived neighborhood safety	Good	4,976	81.0	6,566	79.3	11,542	80.0	5,472	85.7	6,726	83.7	12,198	84.6
	Poor	1,167	19.0	1,711	20.7	2878	20.0	913	14.3	1,307	16.3	2,220	15.4
Natural environment	Good	4,715	76.4	6,287	75.6	11,002	76.0	5,258	82.1	6,426	79.7	11,684	80.8
	Poor	1,456	23.6	2,027	24.4	3483	24.1	1,149	17.9	1,634	20.3	2,783	19.2
Access to public transportation	Good	4,831	78.4	6,497	78.2	11,328	78.3	5,234	81.6	6,511	80.7	11,745	81.1
	Poor	1,329	21.6	1,815	21.8	3144	21.7	1,179	18.4	1,561	19.3	2,740	18.9

Among the participants before the COVID-19 pandemic, 43.3% were males (12,559) and 56.7% were females (16,459). Walking was reported by 53.8% of individuals, with 55.3% of males and 52.6% of females participating. Individuals aged 50–64 years comprised the largest proportion of the study population (29.9%), followed by those aged 35–49 years (23.4%), 65–74 years (17.4%), 19–34 years (17.4%), and ≥75 years (11.9%). Additionally, 40.7% of the participants had completed university education, 29.5% had completed high school, and 29.8% had completed junior high school. Regarding household income, most males and females were within the fourth (23.1%) and first (23.3%) quintiles, respectively. In terms of marital status, 63.2% of participants were partnered.

Additionally, 34.0% of participants reported good subjective health status for healthy behaviors and health status factors; 29.0% had obesity, and 7.2% reported experiencing depression. In 70.3% of participants, there were no chronic diseases, while 23.1% had one, and 6.6% had two or more. This distribution was similar between males and females. Regarding smoking status, 16.9% were current smokers (34.8% male and 3.2% female). For alcohol consumption, 53.4% were current drinkers, including 69.3% of males and 41.2% of females.

Regarding subjective community perceptions, 64.2% of participants reported high trust among neighbors, 80.0% reported high perceived neighborhood safety, 76.0% were satisfied with the natural environment (e.g., air and water quality), and 78.3% were satisfied with access to public transportation. These perceptions were similar among males and females.

Following the COVID-19 pandemic, 44.0% (12,750) of the participants were male, and 56.0% (16,260) were female. The overall walking rate was 44.3%, including 48.0% of males and 41.5% of females. The largest proportion of participants was 50–64 years old (29.6%), followed by those aged 35–49 (22.7%), 65–74 (18.0%), 19–34 (17.7%), and ≥75 years (12.0%). Additionally, 43.4% of the participants had completed university education, 29.4% were high school graduates, and 27.2% were junior high school graduates. Regarding household income, most males and females were within the fourth (24.0%) and first (26.8%) quintiles, respectively. In terms of marital status, 59.1% of the participants were partnered.

A total of 43.3% of participants reported a good subjective health status, indicating healthy behaviors and health status factors. The prevalence of obesity was 28.9%, and 7.2% of participants reported depression. In 70.3% of participants, there were no chronic diseases, 23.3% had one, and 6.5% had two or more. A similar distribution was observed in males and females. In terms of smoking status, 16.0% of the participants were current smokers, including 32.6% of males and 2.9% of females. In terms of alcohol consumption, 46.4% of participants were current drinkers (61.6% of males and 34.5% of females).

Regarding subjective perceptions of the community, 63.5% of the participants reported high trust among neighbors, 84.6% reported high perceived neighborhood safety, 80.8% expressed satisfaction with the natural environment (e.g., air and water quality), and 81.1% were satisfied with access to public transportation. These perceptions were similar for males and females.

Differences in individual characteristics according to walking practice were compared between the before- (*n* = 29,018) and after-COVID-19 (*n* = 29,010) periods. The following results were derived based on variables that showed statistically significant differences between the walking and non-walking groups (Table 2).

**Table 2:** Walking practice-related characteristics of study participants before and after the COVID-19 pandemic.

Variable		Before COVID-19 (2018–2019)						After COVID-19 (2020–2021)					
		No		Yes		χ^2^	*P*-value	No		Yes		χ^2^	*P*-value
		*n*	%	*n*	%			*n*	%	*n*	%		
Total		13,413	46.2	15,605	53.8			16,149	55.7	12,861	44.3		
Sociodemographic factors													
Sex	Male	5,616	44.7	6,943	55.3	20.2056	<.001	6,636	52.1	6,114	48.0	120.7864	<.001
	Female	7,797	47.4	8,662	52.6			9,513	58.5	6,747	41.5		
Age	19–34	2,203	43.6	2,848	56.4	151.974	<.001	2,871	56.1	2,250	43.9	146.7159	<.001
	35–49	3,358	49.5	3,422	50.5			3,877	58.8	2,718	41.2		
	50–64	3,894	44.9	4,789	55.2			4,634	53.9	3,958	46.1		
	65–74	2,124	42.0	2,928	58.0			2,629	50.3	2,599	49.7		
	≥75	1,834	53.1	1,618	46.9			2,138	61.5	1,336	38.5		
Education level	Junior high school	4,030	46.6	4,610	53.4	5.2003	.074	4,448	56.4	3,441	43.6	21.4464	<.001
	High school	3,873	45.2	4,697	54.8			4,573	53.6	3,961	46.4		
	University	5,505	46.7	6,292	53.3			7,122	56.6	5,452	43.4		
Household income	Q1	2,718	48.7	2,863	51.3	61.1529	<.001	3,526	57.8	2,576	42.2	26.3525	<.001
	Q2	2,381	43.2	3,128	56.8			2,716	53.9	2,321	46.1		
	Q3	2,463	44.0	3,134	56.0			2,239	53.3	1,963	46.7		
	Q4	2,689	45.9	3,166	54.1			3,159	55.7	2,517	44.3		
	Q5	2,005	49.4	2,055	50.6			2,130	55.1	1,735	44.9		
Marital status	Partnered	8,515	46.5	9,815	53.6	1.1147	<.001	9,417	54.9	7,733	45.1	9.6453	.002
	Single	4,891	45.8	5,785	54.2			6,728	56.8	5,127	43.3		
Healthy behavior and health status factors													
Subjective health status	Good	3,996	40.5	5,877	59.5	198.8490	<.001	6,558	52.2	6,001	47.8	106.6881	<.001
	Poor	9,416	49.2	9,728	50.8			9,590	58.3	6,860	41.7		
Obesity	Yes	3,882	47.3	4,327	52.7	8.5731	.003	4,774	57.1	3,590	42.9	9.9882	.002
	No	9,132	45.4	10,992	54.6			11,316	55.0	9,243	45.0		
Depression	Yes	1,119	53.8	962	46.2	51.3532	<.001	1,303	62.6	777	37.4	44.2520	<.001
	No	12,294	45.6	14,641	54.4			14,842	55.1	12,083	44.9		
Number of chronic diseases	0	9,324	45.7	11,074	54.3	14.1977	.001	11,259	55.3	9,117	44.7	15.5320	<.001
	1	3,130	46.7	3,573	53.3			3,756	55.7	2,989	44.3		
	Above 2	958	50.1	955	49.9			1,131	60.0	755	40.0		
Smoking	Yes	2,257	46.1	2,636	53.9	0.0218	.883	2,442	52.8	2,187	47.3	18.9389	<.001
	No	11,156	46.2	12,969	53.8			13,706	56.2	10,673	43.8		
Alcohol consumption	Yes	7,014	45.3	8,468	54.7	11.3115	.001	7,176	53.3	6,286	46.7	56.7546	<.001
	No	6,399	47.3	7,136	52.7			8,973	57.7	6,575	42.3		
Subjective perception of the community factors													
Trust among neighbors	Good	4,311	47.6	4,742	52.4	21.8470	<.0001	4,692	52.2	4,300	47.8	3.7221	.054
	Poor	2,607	51.7	2,433	48.3			2,783	53.9	2,384	46.1		
Perceived neighborhood safety	Good	5,630	48.8	5,912	51.2	2.9124	.088	6,370	52.2	5,828	47.8	8.7569	.003
	Poor	1,455	50.6	1,423	49.4			1,235	55.6	985	44.4		
Natural environment	Good	5,435	49.4	5,567	50.6	1.3005	.254	6,160	52.7	5,524	47.3	0.2204	.639
	Poor	1,682	48.3	1,801	51.7			1,481	53.2	1,302	46.8		
Access to public transportation	Good	5,482	48.4	5,846	51.6	11.7298	.001	6,105	52.0	5,640	48.0	16.7474	<.001
	Poor	1,630	51.8	1,514	48.2			1,543	56.3	1,197	43.7		

The walking rate decreased from 53.8% before COVID-19 to 44.3% after the onset of the COVID-19 pandemic. Among the sociodemographic factors, walking practice was lower among females (52.6%) than among males (55.3%) before the COVID-19 pandemic; this sex disparity became more pronounced after the COVID-19 pandemic (*P* < .001). Walking practice rates differed significantly with age (*P* < .001). The highest rates were observed among individuals aged 65–74 (58.0% before and 49.7% after the COVID-19 pandemic; *P* < .001), while the lowest rates were reported among those aged ≥ 75 (46.9% before and 38.5% after; *P* < .001). Regarding the educational level, no significant differences were observed before COVID-19. However, after the COVID-19 pandemic, individuals with at least a high school education showed the highest level of walking (46.4%, *P* < .001). Regarding household income, the third income quintile (Q3) showed the highest walking practices in both periods, with statistically significant differences (*P* < .001). Regarding marital status, single individuals exhibited a higher walking rate before the COVID-19 pandemic (54.2%; *P* < .001). In contrast, after the COVID-19 pandemic, partnered individuals demonstrated higher walking rates (45.1%, *P* = .002).

Among the healthy behavior and health status factors, individuals reporting good subjective health status were likelier to practice walking before and after the COVID-19 pandemic (*P* < .001). Participants with obesity showed lower levels of walking practice; this pattern was more pronounced after the COVID-19 pandemic (*P* = .002). Similarly, individuals experiencing depression demonstrated lower walking rates, with a more prominent difference after the COVID-19 pandemic (*P* < .001).

A negative association was observed between the number of chronic diseases and walking practice: as the number of chronic diseases increased, walking practice decreased, with consistent results in both periods (*P* < .001). Smoking was not significantly associated with walking practice before the COVID-19 pandemic; however, after the COVID-19 pandemic, smokers showed substantially higher rates of walking practice (47.3%, *P* < .001). Alcohol consumption was positively associated with walking, with consistent results before and after the COVID-19 pandemic (*P* < .001).

Regarding subjective perceptions of the community, before the COVID-19 pandemic, individuals who reported higher levels of trust among neighbors were more likely to engage in walking (52.4%, *P* < .001). However, this association lost significance after the pandemic. Perceived neighborhood safety did not significantly impact walking practices before the COVID-19 pandemic; however, after the pandemic, individuals who perceived their neighborhoods as safe were more likely to engage in walking practices (47.8%, *P* = .003). Perceived access to public transportation was positively associated with walking practices during both periods (*P* < .001). However, no significant association was detected between the perceived natural environment quality and walking practices in either period.

### Descriptive statistics of community-level variables

A descriptive analysis of community-level variables revealed that, overall, community environmental factors exhibited positive changes after the COVID-19 pandemic (Table 3).

**Table 3:** Descriptive Statistics of Community-Level Variables Before and After the COVID-19 Pandemic.

Variable	Before COVID-19 (2018 ~ 2019)					After COVID-19 (2020 ~ 2021)				
	*n*	M	SD	Min.	Max.	*n*	M	SD	Min.	Max.
Urban parks (km^2^)	29,018	2.93	3.50	0.07	12.83	29,010	3.71	5.29	0.10	18.95
Pedestrian paths (km)	27,201	32.55	17.31	8.29	66.26	27,192	39.19	18.19	9.50	71.70
Public sports Facilities (count)	29,018	11.75	7.55	1.00	31.00	29,010	15.12	8.30	5.00	40.00
Private sports Facilities (count)	29,018	138.45	65.39	47.00	268.00	29,010	166.05	81.81	46.00	322.00
Social network Difficulty (score)	29,018	1.44	0.22	1.08	1.99	29,010	1.64	0.13	1.38	1.91

Note: SD = standard deviation.

The average area of urban parks increased from 2.93 km2 (standard deviation [SD] = 3.50) before the COVID-19 pandemic to 3.71 km2 (SD = 5.29) afterward. Similarly, the average length of pedestrian paths extended from 32.55 km (SD = 17.31) to 39.19 km (SD = 18.19). The average number of public sports facilities rose from 11.75 (SD = 7.55) to 15.12 (SD = 8.30), and the number of private sports facilities increased from 138.45 (SD = 65.39) to 166.05 (SD = 81.81). Meanwhile, the average score reflecting social network difficulties increased from 1.44 (SD = 0.22) before the pandemic to 1.64 (SD = 0.13) after.

### Effects of individual- and community-level factors on walking practices before and after COVID-19

#### Effects before COVID-19.

A multilevel logistic regression analysis was performed to evaluate the effects of individual- and community-level factors on walking practices among urban residents before the COVID-19 pandemic (Table 4).

**Table 4:** Effects of individual- and community-level factors on walking practice before the COVID-19 pandemic.

Variable	Before COVID-19 (2018 ~ 2019)			
	Model 1	Model 2	Model 3	Model 4
	OR (95% CI)	OR (95% CI)	OR (95% CI)	OR (95% CI)
Fixed effects				
Intercept	1.17^*^ (1.01-1.35)	1.09 (0.82-1.47)	2.24 (0.54-9.26)	0.85 (0.09-7.93)
Individual-level factors (Level 1)				
Socio-demographic factors				
Sex (ref. Male)				
Female	–	0.89^**^ (0.82-0.97)	–	0.89^**^ (0.81-0.97)
Age (ref.19 ~ 34)				
35 ~ 49	–	0.91 (0.80-1.04)	–	0.92 (0.81-1.06)
50 ~ 64	–	0.95 (0.83-1.09)	–	0.96 (0.83-1.11)
65 ~ 74	–	1.16 (0.98-1.38)	–	1.19 (0.99-1.42)
Above 75	–	0.77^**^ (0.64-0.93)	–	0.80^*^ (0.66-0.97)
Education level (ref. Junior high school)				
High school	–	1.03 (0.93-1.15)	–	1.04 (0.93-1.17)
University	–	0.90 (0.79-1.02)	–	0.90 (0.79-1.02)
Household income (ref. Q1)				
Q2	–	1.02 (0.91-1.15)	–	1.07 (0.95-1.21)
Q3	–	1.05 (0.92-1.20)	–	1.10 (0.96-1.26)
Q4	–	1.00 (0.87-1.14)	–	1.05 (0.91-1.21)
Q5	–	0.85^*^ (0.74-0.99)	–	0.85^*^ (0.73-0.99)
Marital status (ref. Single)				
Partnered	–	1.01 (0.92-1.10)	–	0.98 (0.90-1.08)
Healthy behavior and health status factors				
Subjective health status (ref. Poor)				
Good	–	1.36^***^ (1.25-1.47)	–	1.40^***^ (1.28-1.52)
Obesity (ref. No)				
Yes	–	0.96 (0.88-1.04)	–	0.96 (0.89-1.05)
Depression (ref. No)				
Yes	–	0.84^*^ (0.73-0.97)	–	0.83^*^ (0.71-0.97)
Number of chronic diseases (ref. 0)				
1	–	0.99 (0.89-1.09)	–	0.99 (0.90-1.10)
Above 2	–	0.94 (0.81-1.10)	–	0.93 (0.79-1.08)
Smoking (ref. No)				
Yes	–	0.96 (0.86-1.08)	–	0.97 (0.87-1.09)
Alcohol consumption (ref. No)				
Yes	–	1.02 (0.94-1.10)	–	1.01 (0.93-1.10)
Subjective perception of the community factors				
Trust among neighbors (ref. Poor)				
Good	–	1.14^**^ (1.05-1.24)	–	1.16^**^ (1.07-1.27)
Perceived neighborhood safety (ref. Poor)				
Good	–	0.99 (0.89-1.10)	–	0.99 (0.89-1.11)
Natural Environment (ref. Poor)				
Good	–	0.92 (0.83-1.01)	–	0.92 (0.83-1.01)
Access to public transportation (ref. Poor)				
Good	–	1.05 (0.95-1.16)	–	1.06 (0.96-1.16)
Community level factors (Level 2)				
Urban parks	–	–	0.97 (0.92-1.03)	0.94 (0.85-1.03)
Pedestrian paths	–	–	0.99 (0.98-1.01)	1.00 (0.98-1.02)
Public sports facilities	–	–	1.00 (0.97-1.03)	1.02 (0.97-1.06)
Private sports facilities	–	–	1.00 (1.00-1.00)	1.00 (0.99-1.00)
Social network difficulty	–	–	0.86 (0.40-1.85)	1.31 (0.40-4.34)
Variables	Model 1	Model 2	Model 3	Model 4
Random effects				
Between region variance (τ_00_) (SD)	0.11(0.33)	0.16(0.40)	0.06(0.24)	0.14(0.37)
ICC	0.03	0.05	0.02	0.04
-2 log likelihood (deviance)	39483.11	16675.35	36954.43	15563.13
LR Test (chibar^2^)	578.64^***^	408.70^***^	323.90^***^	334.59^***^

Note: OR = odds ratio; CI = confidence interval; Ref = reference; ICC = intraclass correlation; LR test = likelihood ratio test based on chibar² statistic.

Model 1 = null model; Model 2 = included individual-level variables; Model 3 = included community-level variables; Model 4 = included both individual- and community-level variables.

*** p < .001, * p < .01, * p < .05.

Model 1 did not include individual- or community-level independent variables, accounting for only the clustered structure of the data across regions. This null model assessed whether significant differences existed between region in walking practice. The between-region variance in walking practice (τ_₀₀_) was 0.11, corresponding to an ICC of 0.03, indicating that ~3% of the total variance in walking practices was attributable to differences at the community level. Despite being below the 0.05 threshold for meaningful between-group variation [50], multilevel modeling remains useful even with modest ICC values [24]. Therefore, considering community-level variance in this analysis is appropriate, justifying the application of a multilevel modeling approach in this study. Model 1’s fit, evaluated using −2LL, was 39,483.11. The LR test showed that the multilevel logistic regression model had a significantly better fit than the single-level model (*P* < .001).

Among the sociodemographic factors, females had significantly lower odds of walking than males (OR = 0.89; 95% CI: 0.82–0.97). Adults aged ≥ 75 years showed reduced odds of walking compared to those aged 19–34 years (OR = 0.77; 95% CI: 0.64–0.93). Individuals in the highest household income quintile (Q5) had lower odds of walking than those in the lowest quintile (Q1) (OR = 0.85; 95% CI: 0.74–0.99). Regarding healthy behavior and health status, participants who reported good subjective health status were likelier to engage in walking than those with poor subjective health status (OR = 1.36; 95% CI: 1.25–1.47). Those with depression were less likely to engage in walking than those without depression (OR = 0.84; 95% CI: 0.73–0.97). Regarding subjective perceptions of the community, individuals who perceived high levels of trust among neighbors were likelier to engage in walking practice than those with lower perceived trust (OR = 1.14; 95% CI: 1.05–1.24). The between-region variance in Model 2 was τ_00 _= 0.16, with an ICC of 0.03 and −2LL value of 16,675.35, indicating an improvement in model fit compared to Model 1 (−2LL = 39,483.11). The LR test further confirmed that the multilevel model had a significantly better fit than the single-level logistic regression model (Chibar ² (01) = 408.70, *P* < .001).

Model 3 revealed no statistically significant relationships between community-level factors and walking practices. The between-region variance in Model 3 was τ_00 _= 0.06, while the ICC was 0.02 and −2LL was 36,954.43, indicating no improvement in model fit compared to Model 2 (−2LL = 16,675.35). Nevertheless, the LR test showed that the multilevel model provided a significantly better fit than the single-level logistic regression model (*P* < .001).

In Model 4, several individual-level sociodemographic factors were significantly associated with walking practice. Compared with males, females had lower odds of walking (OR = 0.89; 95% CI: 0.81–0.97). Older adults aged ≥ 75 years had reduced odds compared to those aged 19–34 years (OR = 0.80; 95% CI: 0.66–0.97). Individuals in the highest household income quintile (Q5) had lower odds of walking than those in the lowest quintile (Q1) (OR = 0.85; 95% CI: 0.73–0.99). Regarding healthy behavior and health status, participants with good subjective health status were likelier to engage in walking practice than those with poor subjective health status (OR = 1.40; 95% CI: 1.28–1.52). Participants with depression were less likely to engage in walking practice than those without depression (OR = 0.83; 95% CI: 0.71–0.97). Regarding subjective perceptions of the community, individuals who perceived higher levels of neighborhood trust were likelier to engage in walking practice than those with lower perceived trust (OR = 1.16; 95% CI: 1.07–1.27). However, none of the community-level variables were significantly associated with walking. The between-region variance for Model 4 was τ_00 _= 0.14, with an ICC of 0.04 and −2LL of 15,563.13, indicating an improved model fit compared to Model 3 (−2LL = 36,954.43). The LR test further confirmed that the multilevel model provided a significantly better fit than the single-level logistic regression model (*P* < .001).

#### Effects after COVID-19.

The effects of individual- and community-level factors on walking practices among urban residents after the COVID-19 pandemic were evaluated using multilevel logistic regression models (Table 5).

**Table 5:** Effects of individual- and community-level factors on walking practice after the COVID-19 pandemic.

Variable	After COVID-19 (2020 ~ 2021)			
	Model 1	Model 2	Model 3	Model 4
	OR (95% CI)	OR (95% CI)	OR (95% CI)	OR (95% CI)
Fixed effects				
Intercept	0.80^***^ (0.73-0.87)	0.65^**^ (0.50-0.85)	6.82^***^ (3.23-14.36)	6.22^*^ (1.01-38.50)
Individual level factors (Level 1)				
Socio-demographic factors				
Sex (ref. Male)				
Female	–	0.81^***^ (0.75-0.88)	–	0.81^***^ (0.74-0.88)
Age (ref.19 ~ 34)				
35 ~ 49	–	0.91 (0.80-1.03)	–	0.94 (0.82-1.08)
50 ~ 64	–	1.15^*^ (1.00-1.31)	–	1.14 (1.00-1.31)
65 ~ 74	–	1.39^***^ (1.18-1.64)	–	1.40^***^ (1.18-1.66)
Above 75	–	0.91 (0.76-1.09)	–	0.91 (0.75-1.09)
Education level (ref. Junior high school)				
High school	–	1.01 (0.91-1.13)	–	1.01 (0.90-1.13)
University	–	0.94 (0.83-1.07)	–	0.94 (0.83-1.07)
Household income (ref. Q1)				
Q2	–	1.08 (0.96-1.21)	–	1.09 (0.97-1.23)
Q3	–	1.16^*^ (1.02-1.32)	–	1.17^*^ (1.03-1.34)
Q4	–	1.00 (0.88-1.13)	–	0.99 (0.87-1.13)
Q5	–	1.03 (0.90-1.19)	–	1.04 (0.90-1.20)
Marital status (ref. Single)				
Partnered	–	1.04 (0.95-1.13)	–	1.04 (0.95-1.14)
Healthy behavior and health status factors				
Subjective health status (ref. Poor)				
Good	–	1.33^***^ (1.23-1.44)	–	1.32^***^ (1.22-1.43)
Obesity (ref. No)				
Yes	–	0.89^**^ (0.82-0.97)	–	0.90^*^ (0.82-0.98)
Depression (ref. No)				
Yes	–	0.74^***^ (0.64-0.84)	–	0.74^***^ (0.64-0.85)
Number of chronic diseases (ref. 0)				
1	–	0.96 (0.87-1.05)	–	0.96 (0.87-1.06)
Above 2	–	0.85^*^ (0.73-0.99)	–	0.86 (0.73-1.01)
Smoking (ref. No)				
Yes	–	0.94 (0.84-1.05)	–	0.93 (0.82-1.04)
Alcohol consumption (ref. No)				
Yes	–	1.14^**^ (1.05-1.24)	–	1.15^**^ (1.06-1.25)
Subjective perception of the community factors				
Trust among neighbors (ref. Poor)				
Good	–	1.02 (0.94-1.10)	–	1.02 (0.93-1.10)
Perceived neighborhood safety (ref. Poor)				
Good	–	1.11 (0.99-1.24)	–	1.10 (0.98-1.23)
Natural Environment (ref. Poor)				
Good	–	1.00 (0.90-1.10)	–	0.99 (0.89-1.10)
Access to public transportation (ref. Poor)				
Good	–	1.21^***^ (1.09-1.33)	–	1.21^***^ (1.09-1.34)
Community level factors (Level 2)				
Urban parks	–	–	1.00 (0.99-1.02)	1.00 (0.97-1.03)
Pedestrian paths	–	–	0.99^***^ (0.98-0.99)	0.99^**^ (0.98-0.99)
Public sports facilities	–	–	1.01 (1.00-1.01)	1.02^*^ (1.00-1.04)
Private sports facilities	–	–	1.00 (1.00-1.00)	1.00 (1.00-1.00)
Social network difficulty			0.30^***^ (0.20-0.47)	0.28^*^ (0.10-0.80)
Variables	Model 1	Model 2	Model 3	Model 4
Random effects				
Between region variance (τ_00_) (SD)	0.03(0.17)	0.09(0.31)	0.01(0.08)	0.05(0.22)
ICC	0.01	0.03	0.01	0.01
-2 log likelihood (deviance)	39672.80	16517.00	37162.71	15550.34
LR Test (chibar^2^)	170.13^***^	229.07^***^	25.63^***^	97.68^***^

Note: OR = odds ratio; CI = confidence interval; Ref = reference; ICC = intraclass correlation; LR test = likelihood ratio test based on chibar² statistic.

Model 1 = null model; Model 2 = included individual-level variables; Model 3 = included community-level variables; Model 4 = included both individual- and community-level variables.

*** p < .001, * p < .01, * p < .05.

Model 1 indicated that the between-community variance (τ₀₀) in walking practice was 0.03, and the ICC was 0.01, suggesting that approximately 1% of the total variance in walking practices was attributable to differences at the community level. The fit for Model 1 was assessed using −2LL (39,672.80). An LR test compared the fit of the multilevel logistic regression model with that of the conventional single-level logistic regression model. The results indicated that the multilevel model had a significantly better fit (*P* < .001).

In Model 2, several sociodemographic factors were observed to be significantly associated with walking. Females had lower odds of engaging in walking than males (OR = 0.81). Compared to participants aged 19–34 years, those aged 50–64 years (OR = 1.15) and 65–74 years (OR = 1.39) had higher odds of practicing walking. In terms of household income, individuals in the third quintile (Q3) were more likely to walk than those in the lowest quintile (Q1) (OR = 1.16). Regarding healthy behavior and health status, individuals who reported good subjective health were more likely to engage in walking than those with poor subjective health (OR = 1.33). Individuals with obesity had lower odds of walking than those without obesity (OR = 0.89), and those with depression were less likely to walk than those without depression (OR = 0.74). Participants with two or more chronic diseases were less likely to walk than those without chronic diseases (OR = 0.85). Additionally, individuals who reported alcohol consumption were more likely to engage in walking than non-drinkers (OR = 1.14). Regarding the subjective perception of the community, individuals who perceived access to public transportation as good were more likely to engage in walking than those who perceived it as poor (OR = 1.21). The between-region variance in Model 2 was *τ*00 = 0.09, with an ICC of 0.03 and −2LL of 16,517.00, indicating an improvement in model fit compared to Model 1 (−2LL = 39,672.80). The LR test confirmed that the superior fitting of the multilevel model compared to the single-level logistic regression model (*P* < .001).

The two community-level variables were statistically significant in Model 3. A one-unit increase in the length of pedestrian paths was associated with an approximately 1% decrease in the odds of walking (OR = 0.99; 95% CI: 0.98–0.99). Additionally, a one-unit increase in the social network difficulty score was associated with a 70% reduction in the odds of walking (OR = 0.30; 95% CI: 0.20–0.47). The between-region variance in Model 3 was τ_00 _= 0.01, with an ICC of 0.01 and −2LL of 37,162.71, indicating no improvement in model fit compared to Model 2 (−2LL = 16,517.00). However, the LR test showed that the multilevel model provided a significantly better fit than the single-level logistic regression model (*P* < .001).

Several significant associations were identified in Model 4, including individual- and community-level factors. Among the sociodemographic factors, females had lower odds of walking than males (OR = 0.81; 95% CI: 0.74–0.88). Participants aged 65–74 years were more likely to walk than those aged 19–34 years (OR = 1.40; 95% CI: 1.18–1.66). Additionally, individuals in the third income quintile (Q3) had higher odds of walking than those in the lowest (Q1) income quintile (OR = 1.17; 95% CI: 1.03–1.34). Regarding healthy behavior and health status, individuals who reported good subjective health status were likelier to engage in walking than those with poor subjective health status (OR = 1.32; 95% CI: 1.22–1.43). Those with obesity had lower odds of walking than individuals without obesity (OR = 0.90; 95% CI: 0.82–0.98). Participants with depression were less likely to engage in walking practice than those without depression (OR = 0.74; 95% CI: 0.64–0.85). Additionally, individuals who reported alcohol consumption were more likely to engage in walking practice than those who did not consume alcohol (OR = 1.15; 95% CI: 1.06–1.25). Regarding subjective perceptions of the community, participants who perceived access to public transportation as good were more likely to engage in walking practice than those who perceived it as poor (OR = 1.21; 95% CI: 1.09–1.34). Among the community-level factors, a one-unit increase in the length of pedestrian paths was associated with a 1.47% decrease in the odds of walking (OR = 0.99; 95% CI: 0.98–0.99). In contrast, the number of public sports facilities was positively associated with walking practice; a one-unit increase corresponded to a 2.08% increase in the odds of walking practice (OR = 1.02; 95% CI: 1.00–1.04). Additionally, a one-unit increase in the social network difficulty score was associated with a 72.20% decrease in the odds of walking (OR = 0.28, 95% CI: 0.10–0.80). The between-region variance in Model 4 was τ_00 _= 0.05, with an ICC of 0.01 and −2LL of 15,550.34, indicating improved fit compared to Model 3 (−2LL = 37,162.71). The LR test confirmed that the multilevel model fit was superior to the single-level logistic regression model (*P* < .001).

In the single-level logistic regression used for diagnostic purposes, we excluded 426 observations (approximately 1.8% of the sample) that had extreme residuals or high leverage/influence values. When we re-estimated the model after excluding these observations, the overall model fit improved slightly, but the magnitude and statistical significance of the estimated odds ratios changed only marginally compared with the full-sample model. For example, the odds ratio for females compared with males changed from 0.85 to 0.84, with both estimates remaining highly significant (p < .001). These findings indicate that our results are robust to the presence of influential observations.

Diagnostic tests of the linearity-in-the-logit assumption for the continuous district-level covariates (urban parks, pedestrian paths, public sports facilities, private sports facilities, social network difficulty) showed no evidence of important non-linear relationships (all likelihood-ratio test p-values for quadratic terms > 0.05). In addition, models using categorized versions of these variables yielded odds ratios that were highly consistent with those from the main models (data not shown).

## Discussion

This study used SEM [23] to analyze the multilevel determinants of walking practices among 58,028 urban adults in Busan, South Korea, before and after the COVID-19 pandemic. In doing so, this study extends previous research that has typically examined either individual correlates or neighborhood environments at a single time point. By explicitly comparing how individual- and community-level determinants of walking changed across pre- and post-pandemic periods within one multilevel framework, our analysis clarifies which factors became more or less important in the context of COVID-19. Regarding household income, the socioeconomic pattern of walking practice observed in specific sociodemographic groups in Korea is consistent with the high prevalence of insufficient physical activity reported for high-income Asia–Pacific and other OECD regions, where promoting walking and active transport has been identified as a key policy lever for post-pandemic recovery.

Obesity and alcohol consumption, which were not significantly associated with walking practice before the pandemic, became notable factors afterward. Individuals with obesity were less likely to engage in walking practices, whereas alcohol consumers were more likely to do so than non-drinkers. Before the pandemic, higher perceived trust among neighbors was positively associated with walking, whereas after the pandemic, favorable perceptions of public transportation access emerged as a significant facilitator. Females consistently exhibited lower odds of engaging in walking than males [24,26]. This sex disparity widened after the pandemic, potentially attributable to the disproportionate burden of unpaid domestic work and caregiving responsibilities placed on females during the pandemic [51].

Before the pandemic, older adults aged 75 and above were significantly less likely to engage in walking practice compared to younger adults aged 19–35. However, after the pandemic, individuals aged 65–74 years demonstrated a higher likelihood of walking than the younger cohort [24]. This shift suggests that the impact of the pandemic varied across age groups [52].

Regarding household income, individuals in the highest quintile (Q5) were significantly less likely to walk than those in the lowest quintile (Q1) before the pandemic. However, after the pandemic, those in the third quintile (Q3) had a significantly higher likelihood of walking than those in Q1. Although these findings do not reveal a consistent pattern across income levels, they suggest that the impact of the pandemic varied according to socioeconomic group [53].

The positive association between good subjective health status and walking remained relatively high, although it decreased slightly after the pandemic [26]. In contrast, obesity was negatively associated with walking after the pandemic, aligning with previous studies that suggest individuals may develop obesity and become physically inactive during such crises [35]. Additionally, a significant decline in walking was noted among individuals who experienced depression after the pandemic, supporting prior research on the impact of poor mental health on physical activity [21,54]. Meanwhile, a positive association between alcohol consumption and walking was observed only after the pandemic, consistent with findings that light drinking among males and occasional drinking among females are linked to higher levels of walking [55].

Before the pandemic, perceived trust among neighbors was positively associated with walking practices; however, this link weakened after the pandemic. This diminished impact may be due to reduced neighborhood interactions stemming from social distancing and mobility restrictions [56].

Following the pandemic, a positive perception of access to public transportation was significantly associated with walking [24]. This is consistent with the report by Kamelifar et al. [57], which noted that walking and mobility behaviors in the post-pandemic context are closely linked to the urban built environment. At the community level, pedestrian paths, public sports facilities, and social network difficulties were significantly associated with walking practices during the COVID-19 pandemic. However, after the pandemic, pedestrian paths negatively correlated with walking practices, suggesting that qualitative aspects of walking infrastructure, such as perceived safety, accessibility, and usability, are more important than quantitative availability [30]. Moreover, post-pandemic, public sports facilities were positively associated with walking practices, supporting previous findings that community-based sports infrastructures boost physical activity, including walking [29]. In contrast, social network challenges hindered walking, showing that difficulties in maintaining social connections were associated with reduced physical activity levels [24,26,36]. This suggests the pandemic adversely affected social connectedness and physical activity.

Collectively, these findings reinforce the understanding that walking is a multidimensional health behavior shaped by individual, social, and environmental factors. The pandemic has acted as a contextual turning point, altering the relative importance of and the interactions among these factors. Consequently, integrated urban health policies are essential to effectively promote walking practices, particularly those that address individual-level determinants (e.g., sex disparities, mental health) and environmental conditions (e.g., social connectedness, pedestrian infrastructure, and access to public transportation). The findings of this study provide empirical support for walkability-centered urban strategies, such as Busan’s “15-min city” initiative, particularly in post-pandemic or crisis-prone contexts, where equitable access to physical activity opportunities is critical for public health resilience.

### Policy implications for Korea and other Asian cities

The multilevel determinants identified in this study point to concrete strategies for promoting walking practice in Korea and similar high-density Asian cities. At the individual level, tailored walking promotion is needed for groups that showed persistently low walking rates or sharper declines during the pandemic, notably female, adults with obesity, and those reporting depressive symptoms. Primary care and mental health services could incorporate brief walking counselling, step-count monitoring, and referrals to community walking programs as part of routine chronic disease and depression management. Workplace health promotion and digital interventions (e.g., smartphone-based step challenges) may also help middle-aged adults in the third income quintile sustain walking practice under changing work and commuting patterns.

At the interpersonal and community levels, rebuilding social connectedness around walking is critical in the post-pandemic context, where social network difficulties were strongly associated with lower walking. Public health centers, community mental health centers, and local governments could organize neighborhood-based walking groups, women-only or older-adult walking clubs, and “social prescribing” programs that link socially isolated individuals to supervised walking activities in nearby parks and along safe routes. These initiatives should be coordinated with the expansion of public sports facilities documented in Busan, using these facilities as hubs for free or low-cost group walking and exercise programs.

At the environmental and policy levels, our findings suggest that simply increasing the length of pedestrian paths is insufficient and may even be associated with lower walking practice when qualitative aspects of the walking environment are neglected. Urban and transport planners in Busan and other Asian megacities should prioritize the safety, connectivity, and usability of walking routes—such as lighting, barrier-free design, shade and rest areas, and clear way-finding—particularly around public transport stations and in neighborhoods with low walking practice and high social network difficulties. Integrating CHS-based walking practice indicators into local “City of Walking,” “15-minute city,” Healthy City, and age-friendly city policies would enable targeted investments and monitoring. Because many Asian cities share similar characteristics—high residential density, transit-oriented development, and strong gendered care burdens—the multilevel strategies identified in this study can inform region-wide efforts to build physical activity resilience in future pandemics and other crises.

This study has certain limitations. First, the use of cross-sectional data prevented the inference of causal relationships between variables. Second, walking practice was assessed using self-reported CHS items, which are vulnerable to recall and social-desirability bias and do not provide detailed information on walking intensity, duration, or spatial patterns. Future research in Korea and other Asian cities should complement CHS-based self-reported indicators with objective physical activity data from mobile health applications, smartphone step counters, GPS traces, and wearable sensors (e.g., accelerometers and smartwatches) to capture walking behavior more precisely and to examine how specific routes and destinations are shaped by the built environment. Linking such objective data with survey and administrative datasets would allow more advanced multilevel analyses of walking resilience during and after public health emergencies. Third, the analysis excluded several potentially important factors such as occupation, family structure, caregiving burden, and the quality of pedestrian infrastructure. Fourth, certain geographic areas may have been excluded from analyses due to a lack of official data. Fifth, while individual- and community-level factors were considered, interaction effects between these levels were not examined. In addition, we specified random-intercept models with fixed slopes for all covariates, which assume that the effects of individual-level factors are constant across districts. Alternative specifications, such as random-slope or district fixed-effects models, may capture additional heterogeneity in multilevel determinants of walking and should be explored in future research. Finally, as this study focused on a single metropolitan area (Busan), caution is warranted when generalizing the findings to other contexts. Future research should adopt longitudinal designs to better understand the short-term changes induced by COVID-19 and the long-term behavioral trends for more generalizable and robust outcomes.

### Originality and contribution

This study contributes to the extant literature on walking, COVID-19, and urban health in three main respects. First, it moves beyond cross-sectional descriptions of walking during the pandemic by directly comparing multilevel determinants of walking practice before (2018–2019) and after (2020–2021) COVID-19 among Korean urban adults. Second, by linking CHS microdata with administrative and open-government datasets on urban parks, pedestrian paths, public and private sports facilities, and social network difficulty, the study demonstrates how physical infrastructure and social connectedness jointly shape walking under crisis conditions, complementing earlier work that relied primarily on self-reported neighborhood characteristics. Third, by situating the analysis in Busan—a city that has adopted “City of Walking” and “15-min city” strategies—the study provides empirically grounded, policy-relevant evidence that can guide the design of walkability-oriented urban health interventions in other dense metropolitan settings facing current or future public health emergencies.

## Conclusion

This study provides empirical evidence that the COVID-19 pandemic has significantly influenced walking practices among urban adults and emphasizes the importance of individual- and community-level determinants. From a socio-ecological perspective, fostering walkable environments and supportive social networks is critical to sustaining physical activity behaviors such as walking. Community-level factors, including access to pedestrian infrastructure and sports facilities, help maintain walking practices under pandemic-related restrictions.

These findings underscore the need for comprehensive, multilevel interventions that integrate structural, social, and individual strategies. To promote walking as a sustainable health behavior, it is essential to (1) improve the physical environment and associated policies to make walking more convenient and appealing; (2) foster a culture that supports healthy behaviors; and (3) enhance individual engagement through education and motivation. In Korea, these multilevel strategies can be implemented through existing frameworks such as the National Health Plan 2030, the City of Walking initiative, and 15-minute city projects, with priority given to districts and population groups that experienced marked declines in walking during the pandemic. More broadly, other densely populated Asian cities with similar transit-oriented and high-rise urban forms can adapt these strategies to strengthen physical activity resilience against future public health emergencies.

### Consent for publication

The dataset analyzed in this study did not disclose any personally identifiable information. Therefore, consent for publication of this manuscript was not required.

## Supporting information

S1 Table Definitions, coding, and measurement levels of study variables. (DOCX)
