# Supplementary material for: The impact of COVID-19 on walking practices in Korea: Policy implications for Urban health and physical activity resilience
Source: PLoS One. 2025 Dec 19;20(12):e0338875. doi: 10.1371/journal.pone.0338875 (PMC12716708; doi:10.1371/journal.pone.0338875)
Supplement: S1 Table — (DOCX) [file pone.0338875.s001.docx]

**S1 Table.** Definitions, coding, and measurement levels of study variables.

| Level | Variable name | Variable definition | Measurement level | Coding / Notes | Data sources |
| --- | --- | --- | --- | --- | --- |
| Dependent  variable | Walking practice | Walking for at least 30 minutes per day on 5 or more days during the past week, with each session lasting at least 10 minutes | Nominal (Binary) | 0 = Nonwalking  1 = Walking | 1 |
| Level 1  (Individual-  Level variables) | Socio-demographic factors | | |  |  |
|  | Sex | Self-reported | Nominal | 0 = Male  1 =Female |  |
|  | Age | Self-reported | Ordinal | 0 = 19–34  1 = 35–49  2 = 50–64  3 = 65–74  4 = Above 75 |  |
|  | Education level | Self-reported | Ordinal | 0 = Junior high school  1 = High school  2 = University |  |
|  | Household income | Categorized into quintiles based on monthly household income (in 10,000 KRW) | Ordinal | 0 = Q1  1 = Q2  2 = Q3  3 = Q4  4 = Q5 |  |
|  | Marital status | Self-reported | Nominal | 0 = Partnered  1 = Single |  |
|  | Healthy behavior and health status factors | | |  |  |
|  | Subjective health  status | Self-reported | Nominal | 0 = Poor  1 = Good |  |
|  | Obesity | Obesity was defined as BMI ≥ 25 kg/㎡ | Nominal | 0 = No  1 = Yes |  |
|  | Depression | Self-reported | Nominal | 0 = No  1 = Yes |  |
|  | Number of chronic  diseases | Number of chronic diseases | Ordinal | 0 = 0  1 = 1  2 = Above 2 |  |
|  | Smoking | Self-reported | Nominal | 0 = No  1 = Yes |  |
|  | Alcohol  consumption | Self-reported | Ordinal | 0 = No  1 = Yes |  |
|  | Subjective perception of the community | | |  |  |
|  | Trust among  neighbors | Q, People in my neighborhood can be trusted. | Nominal | 0 = Poor  1 = Good | 2, 3 |
|  | Perceived  neighborhood  safety | Q, I am satisfied with the overall level of safety in my neighborhood. | Nominal | 0 = Poor  1 = Good |  |
|  | Natural  environment | Q, I am satisfied with the natural environment in my neighborhood. | Nominal | 0 = Poor  1 = Good |  |
|  | Access to public  transportation | Q, I am satisfied with the public transportation conditions in my neighborhood. | Nominal | 0 = Poor  1 = Good |  |
| Level 2  (Community-  Level  variables) | Urban parks | Urban park area | Continuous | Area | 4 |
|  | Pedestrian paths | Length of sidewalks, walking trails, and pedestrian-only roads | Continuous | Length | 5, 6, 7, 8, 9, 10 |
|  | Public sports  facilities | Number of public sports facilities | Continuous | Count | 11, 12 |
|  | Private sports  facilities | Number of private sports facilities | Continuous | Count | 13, 14 |
|  | Social network  difficulty | Q, In the past year, have you experienced difficulties in your social relationships? | Continuous | Score | 15, 16 |
| Note: Data sources.  1. Korea Disease Control and Prevention Agency. (2018–2019). *Community Health Survey*.  2. Korea Disease Control and Prevention Agency. (2019). *Community Health Survey*.  3. Korea Disease Control and Prevention Agency. (2021). *Community Health Survey*.  4. Busan Metropolitan City. (2021). *Basic Statistics: Parks*  5. Busan Metropolitan City. (2019). *Busan Galmaet-gil and Busan Downtown Galmaet-gil*.  6. Busan Metropolitan City. (2021). *Busan Galmaet-gil and Busan Downtown Galmaet-gil*.  7. Busan Metropolitan City. (2019). *National Road Tourism Information Standard Data*.  8. Busan Metropolitan City. (2021). *National Road Tourism Information Standard Data*.  9. Ministry of Land, Infrastructure and Transport. (2019). *Urban Planning Status (Pedestrian-only roads)*.  10. Ministry of Land, Infrastructure and Transport. (2021). *Urban Planning Status (Pedestrian-only roads)*.  11. Ministry of Culture, Sports and Tourism. (2019). *Public Sports Facilities Nationwide (Status of Village Sports Facilities)*.  12. Ministry of Culture, Sports and Tourism. (2021). *Public Sports Facilities Nationwide (Status of Village Sports Facilities)*.  13. Busan Metropolitan City. (2019). *Reported/Registered Sports Facilities*.  14. Busan Metropolitan City. (2021). *Reported/Registered Sports Facilities*.  15. Busan Metropolitan City. (2018). *Community Security Survey*.  16. Busan Metropolitan City. (2021). *Community Security Survey*. | | | | | |
